# Trade-Off between Operating Time and Energy Consumption in Pulsed Electric Field Electrodialysis: A Comprehensive Simulation Study

**DOI:** 10.3390/membranes11010043

**Published:** 2021-01-08

**Authors:** Manuel César Martí-Calatayud, Mario Sancho-Cirer Poczatek, Valentín Pérez-Herranz

**Affiliations:** IEC Group, ISIRYM, Universitat Politècnica de València, Camí de Vera s/n, 46022 València, Spain; masanpoc@etsii.upv.es (M.S.-C.P.); vperez@iqn.upv.es (V.P.-H.)

**Keywords:** electrodialysis, ion transport simulation, pulsed electric field, concentration polarization, ion-exchange membranes, energy optimization, mass transfer

## Abstract

Electrodialysis (ED) has been recently introduced in a variety of processes where the recovery of valuable resources is needed; thus, enabling sustainable production routes for a circular economy. However, new applications of ED require optimized operating modes ensuring low energy consumptions. The application of pulsed electric field (PEF) electrodialysis has been demonstrated to be an effective option to modulate concentration polarization and reduce energy consumption in ED systems, but the savings in energy are usually attained by extending the operating time. In the present work, we conduct a comprehensive simulation study about the effects of PEF signal parameters on the time and energy consumption associated with ED processes. Ion transport of NaCl solutions through homogeneous cation-exchange membranes is simulated using a 1-D model solved by a finite-difference method. Increasing the pulse frequency up to a threshold value is effective in reducing the specific energy consumption, with threshold frequencies increasing with the applied current density. Varying the duty cycle causes opposed effects in the time and energy usage needed for a given ED operation. More interestingly, a new mode of PEF functions with the application of low values of current during the relaxation phases has been investigated. This novel PEF strategy has been demonstrated to simultaneously improve the time and the specific energy consumption of ED processes.

## 1. Introduction

Electrodialysis (ED) is a mature separation process used to obtain concentrated and diluted streams from saline solutions [1]. In ED, an electric field is applied to set ions in motion within an electrochemical cell, where the use of ion-exchange membranes as separators between different cell compartments creates the concentrate and dilute streams. In addition to the most traditional use of ED as a desalination technology, it has recently been used for new applications in processes where valuable ionic species need to be separated or concentrated from a feed solution [2,3,4,5,6]. ED is a modular technology, where temperature changes such as those needed in evaporative processes are avoided. Also, the addition of chemicals is minimal as compared to competing technologies, such as in those involving the use of ion-exchange resins or chemical precipitation. Thus, the successful production of highly selective and conductive ion-exchange membranes has brought the expansion of electrodialysis into food and biotechnological sectors [7,8,9].

Yet, energy consumption continues to be one of the main challenges of the process. A considerable part of the energy usage is associated with the application of an electric field and the evolving cell electrical resistance. Within an electrochemical cell, the solution layers next to the membranes are the parts where the formation of concentration gradients is more acute; hence, where the major changes in electrical resistance are located. The development of concentration gradients is termed concentration polarization in the field of membrane processes [10]. In ED systems, concentration polarization is an intrinsic consequence of membrane selectivity that depends on the level of applied current density. Apart from the increased cell resistance, in some specific membrane-electrolyte systems, this phenomenon can be accompanied by additional detrimental effects, such as the occurrence of undesired pH changes or the formation of precipitates on the membrane surface [11,12]. Consequently, ED has been commonly limited to working at moderate current densities.

A major research line aiming at improving ED processes is the application of new modes of operation. Enhancement of electroconvection by operating at overlimiting currents is widely studied. When high current densities are applied, electrical instabilities emerge in the depleted solution layers and promote an additional supply of ions from the bulk solution toward the membrane surface [13,14]. Another choice is the operation of ED systems with pulsed electric fields (PEF), which alternate periods of current application with pauses, where the intensity of concentration polarization decreases. Several research works have demonstrated that applying PEF modes can lead to decreased energy consumption and mitigation of fouling phenomena [15,16,17,18]. It has also been found that a combination of PEFs and overlimiting currents can induce increased desalination rates, if electroosmotic mixing lasts during the pauses [19,20]. 

The choice of the parameters of the pulse functions has a paramount influence on the efficiency of this operation mode because formation and relaxation of concentration profiles near ion-exchange membranes is a dynamic process. Previous experimental works have shown that the duration of the pulse and pause periods under different current density regimes can lead to significant differences in terms of the number of charges transported, energy consumption or fouling mitigation [21,22,23,24]. However, the empirical investigation and selection of PEF parameters can result in a time- and resource-consuming practice. The use of flexible but rather simple models to simulate and predict the outcome of applying different pulse shapes can be extremely advantageous in this optimization process. In the present work, we conduct a comprehensive investigation of the effects of different pulse parameters on the energy and operating time needed to obtain a given charge transport in ED systems. A finite difference method applied to a simplified ion transport model is used to evaluate the interplay between the duration and level of applied current during the pulse and pause periods, when PEFs are implemented in a system formed by a cation-exchange membrane and NaCl solutions. In our opinion, the power of this strategy resides not only on a more efficient optimization procedure, but also on the access to information otherwise difficult to obtain experimentally. 

## 2. Model and Simulation

### 2.1. System Geometry and Characteristics

**Figure 1:**
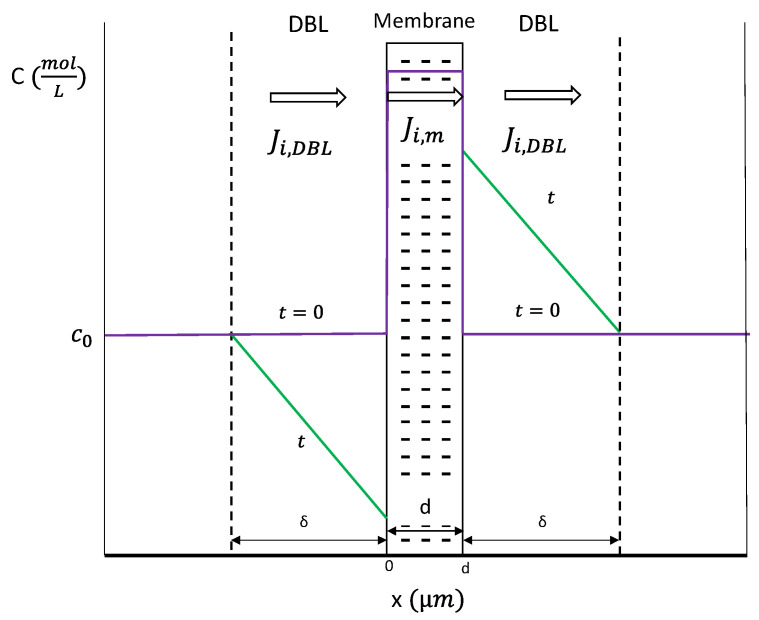
Scheme of the system simulated formed by a cation-exchange membrane surrounded by two diffusion boundary layers and portions of bulk solution. Concentration profiles are represented in the y-axis versus the spatial coordinate *x* for the initial and steady-state conditions.

A membrane-solution system consisting of a homogeneous cation-exchange membrane surrounded by a NaCl solution was simulated with a one-dimensional model using a finite difference method. The geometry of the system is shown schematically in Figure 1, where five differentiated regions can be distinguished:

- Left and right bulk solutions;
- Left (dilute) and right (concentrate) diffusion boundary layers;
- Cation-exchange membrane.

The total thickness of the simulated system corresponds to 1 mm of the ED cell. The thickness (Δ) of each diffusion boundary layer (DBLs) in the simulation was 250 μm, which corresponds to a typical value for ED systems [25,26,27]. A membrane thickness (*d*) of 50 μm was considered [28], while the remaining 450 μm correspond to the portion of well-stirred bulk solution taken into account in the computation of the system voltage drop. The electrolyte considered in the present work is 0.05 M NaCl solutions. During the pulse periods, the system operates under galvanostatic mode. By working with current density values (A/m^2^), the results obtained are specific for a membrane area of 1 m^2^. Operation at room temperature of 25 °C was assumed. It must be noted that the results obtained for a membrane system would be analogous for any membrane within the central repeating units of an ED stack. Consequently, this study will be focused on the ion transport taking place through a single ion-exchange membrane.

### 2.2. Model

The flux of ionic species in the system was modeled using an approximation to the Nernst–Planck equation for transport of charged particles:

(1) $$J_{i}\left(x\right)\;=\;-D_{i}\left(\frac{dC_{i}\left(x\right)}{dx}\right)-\frac{z_{i}\cdot F\cdot D_{i}\cdot C_{i}\left(x\right)}{R\cdot T}\left(\frac{d\varphi \left(x\right)}{dx}\right)+C_{i}\left(x\right)\cdot v\left(x\right)$$

where *J_i_*(*x*) represents the total flux of the species *i* at a given position *x* in the spatial coordinate. The first term on the right-hand side of the equation represents the flux of the species *i* caused by diffusion, the middle term represents the flux by migration and the third term corresponds to the transport in the *x* direction caused by convection. In Equation (1), *D_i_*, *C_i_*(*x*), and *z_i_* represent the diffusion coefficient, the concentration, and the valence of ion *i*; while *F*, *R* and *T* represent the Faraday constant, the gas constant and the temperature. *φ*(*x*) and *v*(*x*) refer to the electric potential and the velocity at *x*.

In the bulk solution, far enough from the membrane surface, fluid flow implies that the convective term provides sufficient mixing, so that the concentration can be considered homogeneous in the *x* direction, as shown schematically in Figure 1. On the contrary, in the DBLs (i.e., very thin solution layers at both sides of the membrane surface), the contribution of convection to ion transport can be neglected. Under the application of an electric field, the selectivity of the membrane implies that transport by migration of counterions in the membrane is higher than in the solution, so that concentration gradients are established at both sides of the membrane. Considering a symmetric 1:1 salt (NaCl) and a galvanostatic mode of operation, Equation (1) that models the flux of ions *i* at any position *x* within the DBLs (*J_i,DBL_*(*x*)) can be converted into:

(2) $$J_{i,DBL}\left(x\right)\;=\;-D_{salt}\left(\frac{dC_{i}\left(x\right)}{dx}\right)+\frac{i_{op}\cdot t_{i}}{z_{i}\cdot F}$$

In Equation (2), the convective term is neglected, the coupled transport of anions and cations is considered by using the salt diffusion coefficient (*D_salt_*), *i_op_* refers to the operating current density and *t_i_* represents the transport number of the species *i* in the solution. The model used in the present study considers the ion concentration and ion flux as the variables of the system, while the rest of the terms are assumed constant. 

The membrane is regarded as a dense phase and has a high concentration of fixed charges, so that migration of ions is the only mechanism of ion transport considered in this part of the system. Accordingly, the flux of ions through the membranes (*J_i,m_*(*x*)) can be expressed as a function of the operating current density:

(3) $$J_{i,m}\left(x\right)\;=\;\frac{i_{op}\cdot T_{i}}{z_{i}\cdot F}$$

In Equation (3), *T_i_* refers to the transport number of ions *i* in the membrane phase. To obtain the time evolution of the concentration in the two DBLs, the continuity equation was also considered:

(4) $$\frac{dC_{i}}{dt}\;=\;-\frac{dJ_{i}}{dx}$$

The system of equations is solved for a system formed by a 0.05 NaCl solution and a cation-exchange membrane, with transport number values of Na^+^ and Cl^−^ ions in the membrane phase of *T_Na_^+^* = 0.95 and *T_Cl_*^−^ = 0.05, respectively. The transport number of Na^+^ and Cl^−^ ions in the solution were obtained from the equivalent conductivity data at infinite dilution, resulting in *t_Na_^+^* = 0.396 and *t_Cl_*^−^ = 0.604 [29]. The salt diffusion coefficient (*D_salt_* = 1.611·10^−9^ m^2^/s) was also calculated from the equivalent ionic conductivity data at infinite dilution using the Nernst–Einstein equation [30]. The simulation problem is solved for different functions of operating current considering partitions in time (*t*) and space (*x*) coordinates. Initial and boundary conditions are needed to solve the simulation problem. The initial conditions (*t* = 0) assumed in our work involve the constant concentration of NaCl in both DBLs equal to *C_bulk_* = 0.05 M and an initial flux of ions equal to 0:

(5) $$C_{Na^{+}}\left(x,0\right)=C_{Cl^{-}}\left(x,0\right)=C_{0}=C_{bulk}\;\forall x,$$

(6) $$J_{Na^{+}}\left(x,0\right)\;=\;J_{Cl^{-}}\left(x,0\right)\;=\;0\;\forall x.$$

The system of equations is solved in each DBL starting from the space node located at the membrane solution interface until reaching the boundary with the bulk solution at a distance Δ from the membrane. The boundary conditions at the edges between the DBL and the bulk solution imply that the concentration is equal to *C_bulk_*, while the boundary conditions at both membrane–DBL interfaces are represented by the equality of fluxes at both sides of the interface:

(7) $$C_{Na^{+}}\left(-\delta ,t\right)=C_{Cl^{-}}\left(-\delta ,t\right)=C_{Na^{+}}\left(d+\delta ,t\right)=C_{Cl^{-}}\left(d+\delta ,t\right)=C_{bulk}\;\forall t,$$

(8) $$J_{Na^{+}}\left(0,t\right)=J_{Na^{+}}\left(d,t\right)=J_{Na^{+},m}=\frac{i_{op}\cdot T_{Na^{+}}}{z_{Na^{+}}\cdot F}\;\forall t,$$

(9) $$J_{Cl^{-}}\left(0,t\right)\;=\;J_{Cl^{-}}\left(d,t\right)=J_{Cl^{-},m}=\frac{i_{op}\cdot T_{Cl^{-}}}{z_{Cl^{-}}\cdot F}\;\forall t.$$

Equations (2) and (4) solved in this model are sensitive in terms of stability, so they were converted into dimensionless forms by dividing the variables *c_i_*, *J_i_*, *t_i_* and *x_i_* by reference values *cº_i_*, *Jº_i_*, *tº_i_* and *xº_i_*, according to the rules of Langtangen and Pedersen, where the subindex *i* refers to either Na^+^ or Cl^−^ ions [31]. The system is solved for one spatial dimension, *x*, using the finite difference method, which allows obtaining the profiles of the dependent variables, *J_i_* and *C_i_*, after their conversion in the dimensional forms. The system of equations was applied to each DBL subsystem, while the subsystem membrane and bulk solution can be assumed as constant ohmic resistances. The space and time variables are discretized establishing grids over both domains at space and time steps, Δ*x* and Δ*t*, respectively.

Once the concentration profiles across the system at different times have been obtained, the potential difference between two consecutive spatial nodes (*i*−1 and *i*) can be calculated by using the following equation [27]:

(10) $$\frac{d\varphi }{dx}\;=\;\frac{i_{op}\left(t\right)}{k\left(c\right)}-\frac{R\cdot T}{F}\left(\frac{t_{1}}{z_{1}}+\frac{t_{2}}{z_{2}}\right)\frac{d\;ln\left(c\left(x,t\right)\right)}{dx}$$

which in its integral form for the investigated system can be expressed as:

(11) ΔU(i−1:i,t)=iop(t)·Δx·ln(k(CNaCl(i,t))k(CNaCl(i−1,t)))k(CNaCl(i,t))−k(CNaCl(i−1,t))−R·TF(tNa+−tCl−)ln(CNaCl(i,t)CNaCl(i−1,t)). 

The function $k\left(C_{NaCl}\right)$, which represents the conductivity of NaCl solutions as a function of the salt concentration, was obtained from the interpolation of experimental data reported in the literature [29]. ΔU(i−1:i,t) values are computed between all adjacent nodes of the DBLs and are summed to obtain the voltage drop for the diluted and concentrated DBL subsystems, Δ*U_dil_*(*t*) and Δ*U_conc_*(*t*), respectively. The voltage drop in the portions where a constant resistance is assumed, i.e., the solution bulk and the membrane phase, can be calculated according to Equation (12):

(12) $$\Delta U_{subsystem}\left(t\right)\;=\;\frac{i_{op}\left(t\right)\cdot L}{k_{subsystem}},$$

where *L* represents the thickness of the subsystem and *k_subsystem_* was calculated as *k*(0.05 M) with the interpolated function $k\left(C_{NaCl}\right)$ for the bulk solution, and a characteristic value was taken for the membrane conductivity. The total voltage drop of the membrane system Δ*U_m_* was calculated as the sum of the contributions of all subsystems:

(13) $$\Delta U_{m}\left(t\right)=\Delta U_{bulk}\left(t\right)+\Delta U_{dil}\left(t\right)+\Delta U_{conc}\left(t\right)+\Delta U_{membrane}\left(t\right)$$

Finally, the specific energy consumption (kJ/m^2^) for a given degree of desalination or number of charges transported can be calculated by integrating the product between the voltage drop through the membrane system and the input function of operating current density over time:

(14) $$E\;=\;\int_{t_{0}\;=\;0}^{t_{f}}i_{op}\left(t\right)\cdot \Delta U_{m}\left(t\right)\mathrm{dt}$$

Here, we want to emphasize that this model entails several assumptions and simplifications. For instance, it is assumed that the local electroneutrality condition holds in all parts of the system, and the dissociation of water has not been taken into account. These phenomena may become relevant, especially at overlimiting currents, but are very specific of the membrane and solution properties. Also, the use of 1D models implies neglecting the effects of membrane heterogeneity and electroconvection. However, the agreement between the ohmic regions of current-voltage curves obtained using 1D and 2D models was confirmed by M.K. Urtenov et al. in a previous work [32].

### 2.3. Implementation of PEF Currents

**Figure 2:**
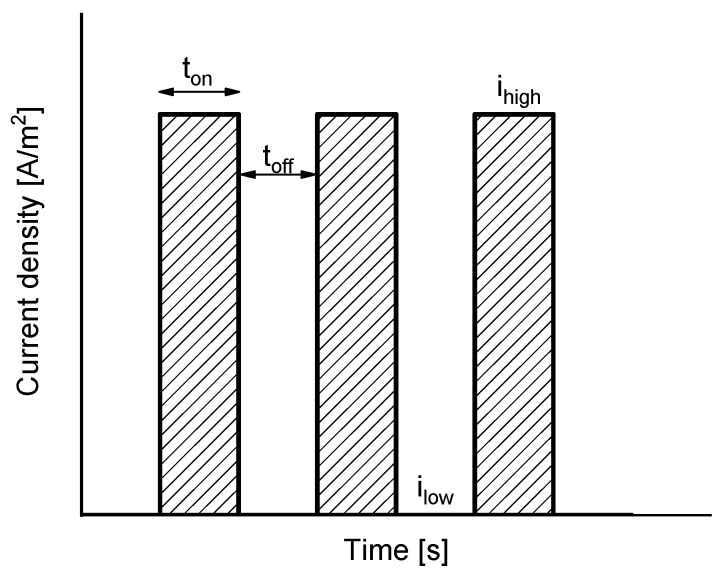
Scheme of the input functions of applied current density.

The calculation of the dynamic system response with the model described above was accomplished for varying PEF input functions of current density, *i_op_*(*t*). As can be seen in Figure 2, the input functions alternate periods where a constant current density is applied with periods of pauses or relaxation of the membrane system, where a zero or low level of current density is applied. Thus, four primary parameters of the input functions were varied in this work: the level of current density applied during the pulses (*i_high_*), the level of current density applied during the pauses or relaxation periods (*i_low_*), the time of pulse duration (*t_on_*) and the time of pause or relaxation duration (*t_off_*). The application of current pulses was also compared with the behavior of the system under galvanostatic mode without pauses.

The variation of the primary pulse function parameters (*i_high_*, *i_low_*, *t_on_*, *t_off_*) was implemented to simulate the system under varying values of frequency and duty cycle, which are parameters more commonly used in the literature to define the characteristics of PEF modes of operation. The frequency of a cycle (*f*) is defined as:

(15) $$f\;=\;\frac{1}{t_{on}+t_{off}}$$

The duty cycle (*α*) correlates the time during which the pulses are applied with the total duration of a cycle:

(16) $$\alpha \;=\;\frac{t_{on}}{t_{on}+t_{off}}.$$

In the following section, a comprehensive investigation of the effect of the frequency and duty cycle of the pulses on the membrane voltage drop at different levels of pulse current density (*i_high_*) is presented. The values of current during the relaxation periods (*i_low_*) were also investigated. It must be noted that the simulation time was adapted in order to compare the results for a value of transported charge equal to 16,800 A·s in all cases.

## 3. Results and Discussion

### 3.1. Results Obtained Applying a Constant Current in Continuous Mode

First, the transport of ions through the membrane system was simulated under the application of a continuous current at different levels, so that these reference conditions could be used for comparison purposes with the PEF modes. Considering the characteristics of the simulated system described in the previous section, the limiting current density can be calculated theoretically by means of the Peers’ equation [33]:

(17) $$i_{lim}=\frac{F\cdot D_{NaCl}\cdot C_{0}}{\delta \cdot \left(T_{Na^{+}}-t_{Na^{+}}\right)}.\;$$

The current density for the membrane-electrolyte system selected for this study corresponds to a value of 56.12 A/m^2^. Table 1 summarizes the parameters considered for the simulations in continuous galvanostatic mode. Apart from the operation at *i_lim_*, two levels of underlimiting current density were also simulated to evaluate the system response at varying degrees of concentration polarization. For evaluation purposes, the application of overlimiting currents was also investigated. Here, it must be noted that at overlimiting currents, mechanisms of mass transfer different from those contemplated in the model come into play. More specifically, the dissociation of water and the onset of electroconvection become relevant when the electrolyte concentration at the membrane–DBL interface approaches zero [34,35,36]. Thus, the results obtained at *i_op_* > *i_lim_* will be considered only for short time spans during which the concentration of species at the depleting membrane–DBL interface is still far from zero. The simulation time was chosen to be 10 min for the 0.5·*i_lim_* case, while it was adapted for the rest of simulations so as to simulate the same amount of charge transported through the membrane. 

**Figure 3:**
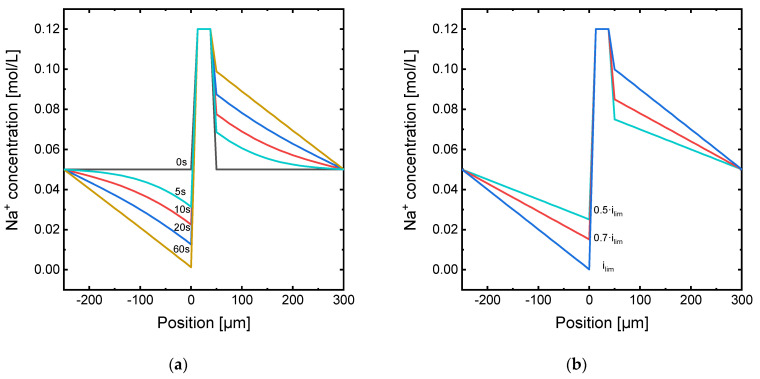
Na^+^ concentration profile along the diffusion boundary layers for the membrane system under: (**a**) application of *i_lim_* at different times; (**b**) application of different current densities once the steady state is achieved.

Figure 3 shows the concentration profiles resulting from the simulation results under the continuous application of current. Note that electroneutrality conditions across the DBLs involve that the concentration profiles for Cl^−^ ions have the same shape as those shown in the figures for Na^+^. Figure 3a shows the evolution with time of the concentration in the two DBLs when a current density equal to *i_lim_* is applied. It can be seen how the concentration of Na^+^ ions decreases gradually with time in the depleting DBL until reaching a value close to zero at the membrane interface after 60 s of current application. On the contrary, in the concentrate compartment, the concentration rises as a consequence of the faster transport of Na^+^ through the membrane as compared with the transport through the DBL towards the cathode. Figure 3b shows the steady-state concentration profile obtained under the application of different levels of current density. As expected, when the steady state is achieved, the fully developed concentration profiles across the Nernst diffusion layer adopt the shape of straight lines. While the Na^+^ concentration at the depleting membrane–DBL interface takes the value of zero for *i_lim_*, this value is significantly higher in the case of underlimiting currents.

**Figure 4:**
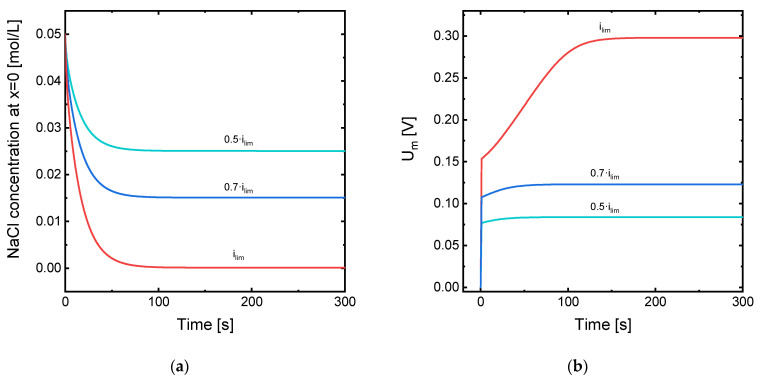
(**a**) Evolution with time of the NaCl concentration at the interface between the membrane surface and the diluting DBL (position *x* = 0) for different levels of applied current density; (**b**) evolution with time of the membrane system potential drop (*U_m_*) for different levels of applied current density.

The evolution with time of the Na^+^ concentration at the depleting membrane-DBL interface for the system under the application of different values of current density is represented in Figure 4a. Here, it can also be seen that the concentration at the membrane surface reaches a value of zero when *i_op_* = *i_lim_* and the steady state is achieved. This result confirms that the simulation is consistent with the Peers’ equation. Figure 4b also shows the evolution of the voltage drop across the membrane system for the three values of applied current. Here, the initial jump in *U_m_* registered at the beginning of the simulation is approximately proportional to the applied current, as it is expected from a quasi-ohmic relationship between current and voltage when the influence of concentration polarization is still small. However, as the time increases, a jump in the *U_m_* value is registered before reaching a stationary value, especially for the *i_lim_* case. The time needed to reach the steady state also increases with the applied current: the value of *U_m_* reaches a constant value at around 50 s when 0.5·*i_lim_* is applied, while it needs more than 100 s in the case of *i_lim_*. From the results obtained with the continuous application of current, we can already expect that the implementation of current pulses will be more beneficial at high current densities, when the concentration profiles in the DBLs are more abrupt and the associated increase in *U_m_* is bigger.

### 3.2. Effect of Frequency

The effect of the frequency of PEF input functions was analyzed at three different levels of current density: 0.7·*i_lim_*, *i_lim_* and 1.2·*i_lim_*. As deduced from the previous subsection, the almost flat evolution of *U_m_* with time for 0.5·*i_lim_* indicates that the benefits of applying pulse current functions may be minor at very low values of current density. In contrast, the results obtained at 1.2·*i_lim_* are expected to be more beneficial in terms of energy savings; although the model can only be used in the unsteady condition for this current, i.e., under the condition that the electrolyte concentration at the depleting membrane–DBL is higher than zero. The duration of the pulses was also selected from the evolution of *U_m_* versus time shown in Figure 4b, considering times before the attainment of the steady state. In this section, the pause duration (*t_off_*) was set to the same value as the pulse duration (*t_on_*), so the values of *α* are 0.5 for all simulations. The pulse function parameters are summarized in Table 2.

**Figure 5:**
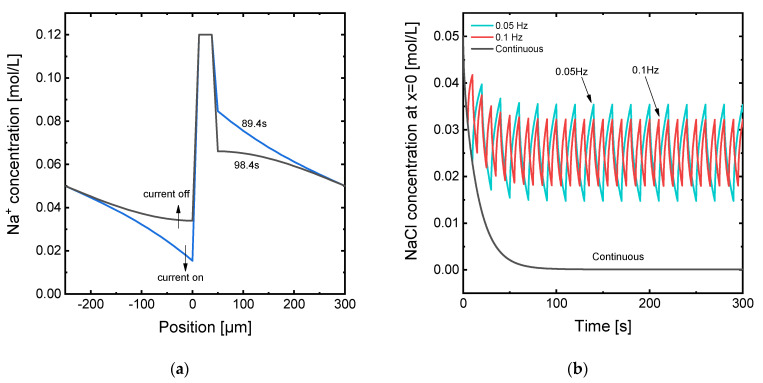
(**a**) Na^+^ concentration profile along the diffusion boundary layers for the membrane system under application of *i_lim_* in pulsed electric field (PEF) mode with a pulse frequency of 0.05 Hz at different times corresponding to pulse and relaxation phases; (**b**) evolution with time of the NaCl concentration at the interface between the membrane surface and the diluting diffusion boundary layer (DBL) (position *x* = 0) under the application of *i_lim_* in continuous mode and in PEF mode at different frequencies. The arrows in the first plot indicate the evolution of concentration profiles during the pulses and relaxation phases.

Figure 5a shows some characteristic concentration profiles at times corresponding to the pulse and pause periods. When the current is on, the concentration drops through the diluting DBL and increases through the concentrating DBL. In both cases the concentration decreases from the anodic side of the electrochemical cell (left part of the graphs) towards the cathodic side (right part of the graphs). The gradient is also sharper near the membrane–DBL interfaces. However, when the current ceases in a pause phase, the relaxation of the concentration gradients is also more notorious near the membrane interfaces. This effect can be related to the driving force for diffusive mass transfer existing in the system right after the application of a current pulse: the differences of concentration are bigger next to the membrane surface, and the diffusive mass transfer between two locations where the differences in concentration are bigger takes place faster. The concentration of Na^+^ at the diluting membrane–DBL interface is also plotted at different frequencies for *i*_op_ = *i_lim_* in Figure 5b. In contrast with the results achieved when the current is continuously applied, the application of pulses promotes an oscillatory evolution of the concentration with time. The most remarkable outcome from the application of pulses is that the concentration does not reach the value of 0 at the membrane interface. Thus, concentration polarization can be effectively attenuated. For a duty cycle of *α* = 0.5, the mean value of concentration at the membrane interface is approximately invariant with the frequency; however, the amplitude of the oscillations becomes smaller as the frequency increases.

**Figure 6:**
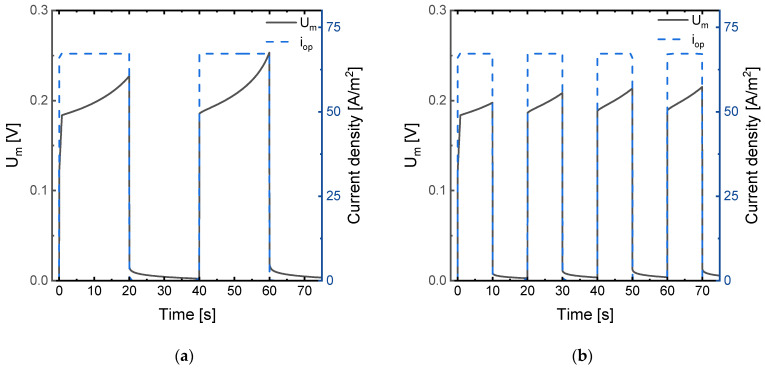
Evolution with time of the membrane system potential drop (*U_m_*) under the application of 1.2·*i_lim_* in PEF mode with *α* = 0.5 and (**a**) pulse frequency *f* = 0.025 Hz (*t_on_* = 20 s, *t_off_* = 20 s); and (**b**) pulse frequency *f* = 0.05 Hz (*t_on_* = 10 s, *t_off_* = 10 s).

A more detailed examination of the evolution of *U_m_* for different frequencies is shown in Figure 6. When the pulse frequency is low (Figure 6a), the duration of the pulse is longer, and *U_m_* reaches higher values during the pulse phase. However, the longer duration of the relaxation phases at lower frequencies implies the restoration of *U_m_* to lower values before the start of a new pulse. Conversely, at high frequencies (Figure 6b), concentration profiles undergo a relaxation process before reaching critical *U_m_* values, so that concentration polarization can be controlled more efficiently.

**Figure 7:**
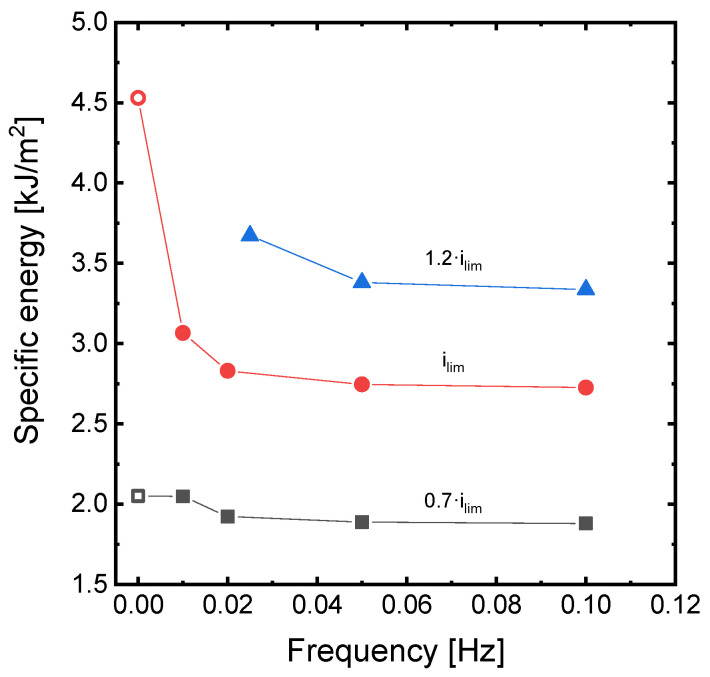
Effect of frequency of PEF implementation on the specific energy consumption at different levels of current density and duty cycle *α* = 0.5. Empty symbols represent the operation in continuous mode.

To provide a global picture of the effect of pulse frequency on the specific energy consumption, the different simulated results are compared in Figure 7. The effect of the application of PEFs is very moderate at 0.7·*i_lim_*, where concentration polarization is not very intense. However, the reduction in energy consumption becomes noticeable when increasing the frequency from 0.01 Hz to a value of 0.02 Hz, and then reaches a plateau at higher frequencies. At current densities equal to *i_lim_*, when the operation is changed to PEF modes, the reduction in specific energy consumption represents more than a third with respect to the value obtained in continuous mode. The biggest change is observed when frequency increases from 0 to 0.01 Hz, and then energy consumption decreases more gradually with frequency until reaching a plateau around 0.05 Hz. Thus, for the investigated system, the application of frequencies higher than 0.05 Hz does not provide a further improvement of the process. For the operation at 1.2·*i_lim_*, similar trends confirm the effect of frequency on the specific energy consumption. However, the minimum value is shifted towards higher frequencies.

### 3.3. Effect of Duty Cycles

The significant reduction in energy consumption observed with the application of PEFs at different frequencies could be further optimized by varying the duty cycle, that is, by implementing asymmetric pulse functions with differing *t_on_* and *t_off_* values. Moreover, the evaluation of the effect of duty cycle on the performance of ED systems may also show improvements, not only related to the energy consumption, but also to the time needed to transport a given number of ions through the membranes. Since in the previous section, the specific energy consumption reached an almost minimum plateau value at frequencies close to 0.05 Hz, this value has been taken as the central one around which the effect of duty cycle is investigated. It must be mentioned that, by varying the duty cycle, the values of frequency also change slightly around the central value of 0.05 Hz. Table 3 summarizes the conditions of current density pulse functions. In essence, in this section, the study is equivalent to varying the pause duration for a fixed pulse duration of 10 s.

**Figure 8:**
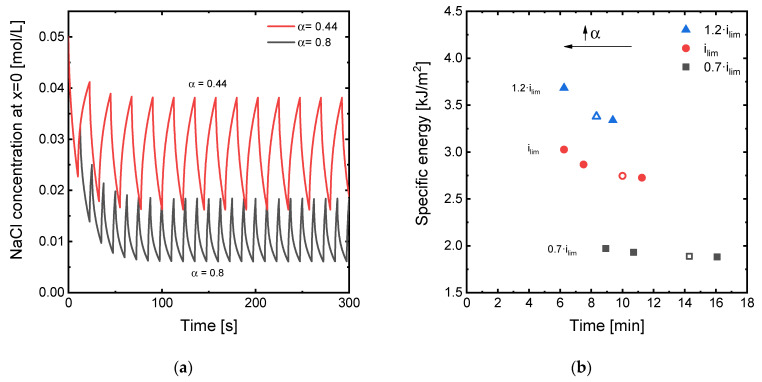
(**a**) Evolution with time of the NaCl concentration at the interface between the membrane surface and the diluting DBL (position *x* = 0) under the application of *i_lim_* in PEF mode with different duty cycles: 10 s–12.5 s (*α* = 0.44) and 10 s–2.5 s (*α* = 0.8); (**b**) trade-off between the specific energy consumption and the time needed to achieve a charge transport of 16,800 A·s at different levels of current density with *t_on_* = 10 s and varying duty cycles. Empty symbols represent the conditions for *α* = 0.5, where *t_on_* = *t_off_* = 10 s.

Some of the most representative results obtained by varying α are presented in Figure 8. For pulse periods with *t_on_* = 10 s, values of *α* higher than 0.5 imply pauses shorter than 10 s, while lower values of *α* imply extending the pause duration. Regarding the evolution of Na^+^ concentration at the diluting membrane–DBL interface (Figure 8a), higher duty cycles lead to a more intense depletion of the DBL, with concentrations at *x* = 0 oscillating around 0.01 mol/L when *i_high_* = *i_lim_*. On the contrary, a lower *α* value of 0.44 leads to Na^+^ concentrations at the membrane–solution interface oscillating around 0.025 mol/L. It can also be seen that the oscillation amplitudes become broader when *α* decreases, which is caused by the longer relaxation times leading to an enhanced restoration of the concentration profiles within the DBLs. Sosa-Fernandez et al. already demonstrated in a previous study, that extending the pause periods (*t_off_* values) during the desalination by PEF ED of polymer-flooding produced water yielded lower energy consumptions [16]. However, this reduction in energy consumption occurs at the cost of extending the time of operation significantly. Figure 8b shows the correlation between specific energy consumption and time needed to achieve a charge transfer of 16,800 A·s for different duty cycles. As found in previous works, decreasing α implies a decrease in the energy consumption, being this reduction more notorious at high current densities. On the contrary, for *α* values higher than 0.5, the times saved by decreasing the pause periods implied an increase in energy consumption. Thus, the application of asymmetric pulses can be used to obtain a trade-off between energy and time needed to desalinate a feed stream, being a useful strategy depending on the specific priorities of the given application of ED.

### 3.4. Effect of the Current Density Applied during the Relaxation Phases

In addition to changing the frequency and duty cycle, which depend on the values of *t_on_* and *t_off_*, and evaluating their effect on the system’s performance at different current density regimes (*i_high_*), an alternative means to reduce the time needed to achieve the transport of a given number of charges through ED membranes is to implement periods of relaxation at currents different from zero. To our knowledge, this type of PEF implementation has not been evaluated before, and it could simultaneously entail improvements in both the time and the energy needed to reach a specific desalination degree. To explore this new type of pulse functions, the simulation with symmetric pulses at frequencies equal to 0.05 Hz (*t_on_* = *t_off_* = 10 s) was performed with values of *i_low_* of 0, 0.25·*i_lim_* and 0.5·*i_lim_*.

**Figure 9:**
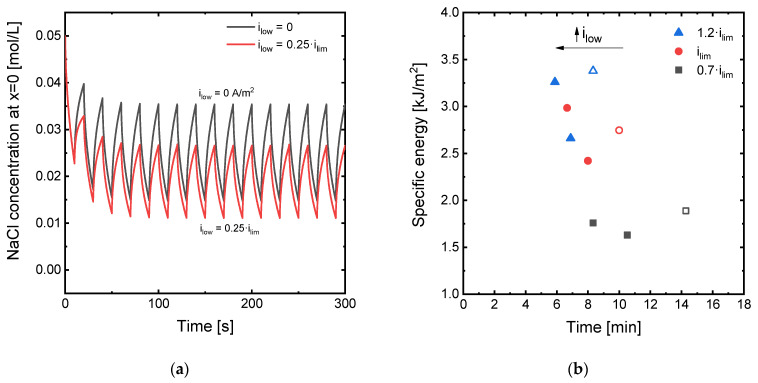
(**a**) Evolution with time of the NaCl concentration at the interface between the membrane surface and the diluting DBL (position *x* = 0) under the application of *i_lim_* in PEF mode with different values of *i_low_* applied during the relaxation periods; (**b**) trade-off between the specific energy consumption and the time needed to achieve a charge transport of 16,800 A·s at different levels of current density with *t_on_* = *t_off_* = 10 s and varying values of *i_low_*. Empty symbols represent the conditions for *i_low_* = 0 A/m^2^.

Figure 9 shows an overview of the simulation results obtained varying *i_low_*. In Figure 9a, the evolution with time of the concentration of Na^+^ ions at the diluting membrane–DBL interface are shown for two different values of *i_low_*. The evolution of the concentration with time clearly shows that applying a value of current density different from zero during the relaxation phases of the PEF functions is effective in attenuating concentration polarization, because the Na^+^ concentration increases during this phase of the input signals. In fact, the shape of the curves is similar to the case where the current is turned off during the pauses. While the oscillations take place at a slightly lower value of concentration, their amplitude is somewhat smaller for values of *i_low_* = 0.25·*i_lim_*. Moreover, during the relaxation phases, current is still being effectively transported through the membranes, which implies a reduction in the operating time. Figure 9b summarizes the results obtained in this section. The application of 0.25·*i_lim_* during the relaxation phases of the pulse functions induce a simultaneous reduction of the time and energy needed to achieve the same number of charges transported through the membranes, thus proving the convenience of adopting this strategy in ED systems. This improvement is general for all values of *i_high_*, but more significant for *i_high_* values close or higher than *i_lim_*. A further increase in the *i_low_* values up to 0.5·*i_lim_* shows to be also beneficial for the time needed for a given desalination objective, but it represents a further increase in the specific energy consumption as compared with the cases where *i_low_* = 0.25·*i_lim_*. These improvements observed in the ED performance by applying low values of current during the relaxation phase of PEF modes are even more notorious when compared with the results obtained operating in continuous mode. For example, for the case when *i_high_* = *i_lim_*, a substantial decrease in specific energy is achieved, changing from 4.53 kJ/m^2^ in the continuous mode to 2.42 kJ/m^2^ in the PEF mode with *i_low_* = 0.25·*i_lim_*. However, the operating time only increases from five to eight minutes, thus representing a significant improvement as compared with conventional PEF modes where the current ceases completely during the relaxation phases.

## 4. Conclusions

In this work, the effect of the input signal parameters on the performance of pulsed electric field electrodialysis has been systematically evaluated using a facile simulation model solved applying finite element methods. Different parameters, such as pulse frequency, duty cycle or applied current density in both the pulse and relaxation phases have been evaluated in terms of the specific energy consumption and operating time needed to achieve a constant transport of charges through the membranes. In general, it can be concluded that changing from continuous to PEF modes implies a general decrease in specific energy consumption; being this improvement more significant at high current densities, where high degrees of polarization are reached. There is a threshold frequency value from which further improvements in energy consumption become almost negligible. This frequency depends on the level of applied current *i_high_*, taking a value of 0.02 Hz for 0.7·*i_lim_* and increasing up to 0.05 Hz for 1.2·*i_lim_*. Decreasing the duty cycle provides moderate savings in energy consumption, while increasing it implies significant reductions in the operating time needed for a given desalination process. For the first time, a novel type of PEF function with application of current during the relaxation periods has been evaluated. These new PEF modes are found to be a promising strategy to effectively reduce the specific energy consumption, without compromising the operating time of ED processes. Application of low levels of current density during the relaxation periods is useful for attenuating concentration polarization, while maintaining ion transport during the entire ED operating time.

## Figures and Tables

**Table 1:** Values of current density, simulation time and transported charge considered for the simulation of continuous galvanostatic electrodialysis (ED) in the present work.

*i_op_* (mA/cm^2^)	Simulation Time (min)	Charge (A·s)
0.5·*i_lim_* = 28.06	10	16,800
0.7·*i_lim_* = 39.28	7.14	16,800
*i_lim_* = 56.12	5	16,800
1.2·*i_lim_* = 67.34	4.17	16,800

**Table 2:** Pulse parameters considered for the investigation of the effect of pulse frequency functions on the ED system.

*t_on_* (s)	*t_off_* (s)	Frequency (Hz)	Duty Cycle	Charge (A·s)
50	50	0.01	0.5	16,800
25	25	0.02		
10	10	0.05		
5	5	0.1		

**Table 3:** Pulse parameters considered for the investigation of the effect of duty cycle on the ED system.

*t_on_* (s)	*t_off_* (s)	Frequency (Hz)	Duty Cycle	Charge (A·s)
10	2.5	0.08	0.8	16,800
	5	0.066	0.66	
	10	0.05	0.5	
	12.5	0.044	0.44	

## Data Availability

No new data were created or analyzed in this study. Data sharing is not applicable to this article.
